# VM-RTDETR: Advancing DETR with Vision State-Space Duality and Multi-Scale Fusion for Robust Pig Detection

**DOI:** 10.3390/ani15223328

**Published:** 2025-11-18

**Authors:** Wangli Hao, Shu-Ai Xu, Hao Shu, Hanwei Li, Meng Han, Fuzhong Li, Yanhong Liu

**Affiliations:** 1 Faculty of Software Technologies, Shanxi Agricultural University, Jinzhong 030801, China; 20233031@stu.sxau.edu.cn (S.-A.X.); 20233028@stu.sxau.edu.cn (H.S.); lifuzhong@sxau.edu.cn (F.L.); 2 College of Information Science and Engineering, Shanxi Agricultural University, Jinzhong 030801, China; 202430801@stu.sxau.edu.cn (H.L.); hanm@hdu.edu.cn (M.H.); 3 Hangzhou Economic Development Zone, Hangzhou Dianzi University, Hangzhou 310018, China

**Keywords:** pig detection, VM-RTDETR, state-space model, multi-scale feature learning, intelligent livestock monitoring

## Abstract

Pig detection is a fundamental yet challenging task in intelligent livestock farming, primarily due to difficulties in capturing both global contextual information and multi-scale features within complex environments. To address this, we propose VM-RTDETR, a novel detection model based on an enhanced RT-DETR architecture. The model incorporates a Vision State-Space Duality (VSSD) backbone, leveraging a novel Non-Causal State-Space Duality (NC-SSD) mechanism to overcome the limitations of traditional SSMs by enabling efficient modeling of long-range dependencies and global context. Furthermore, we design a Multi-Scale Efficient Hybrid Encoder (M-Encoder) that employs parallel convolutional kernels to capture both local details and global contours, effectively addressing scale variations. The synergistic design of the VSSD backbone and the M-Encoder enables our model to achieve more comprehensive feature representation. Experimental results on a custom dataset of 8070 images from a pig farm (with 6955 images for training and 1115 for testing) demonstrate that VM-RTDETR significantly outperforms existing mainstream detectors, improving AP, $AP_{50}$, and $AP_{75}$ by up to 2.35%, 0.63%, and 2.76%, respectively, over the strong R50-RTDETR baseline. Our model significantly enhances detection robustness in complex scenarios, offering an efficient and accurate solution for intelligent livestock farming.

## Simple Summary

Robust pig detection in complex farming environments requires a unified representation of both global semantics and local details, which remains a challenge. This paper proposes VM-RTDETR, an enhanced RT-DETR (transformer-based real-time object detector) model that addresses this by synergizing a Vision State-Space Duality (VSSD) backbone with a Multi-scale Encoder (M-Encoder). The VSSD module breaks through the causal constraints of traditional state-space models (efficiently capturing long-range dependencies and global context within an image) to capture long-range dependencies and global context, while the M-Encoder extracts parallel multi-scale features to handle appearance variations. This collaboration yields a detector that robustly handles scale changes, occlusions, and complex backgrounds. On challenging datasets, VM-RTDETR elevates the state of the art, surpassing strong baselines like RT-DETR by significant margins. It provides a reliable and efficient vision solution for automated livestock monitoring.

## 1. Introduction

Pig detection is a core technology for automated and standardized livestock farming, playing a crucial role in individual animal management, disease monitoring, and optimized resource allocation [1]. However, this task is particularly challenging in complex farm environments. Issues such as occlusions, varying lighting conditions, and overlapping among pigs significantly degrade detection performance [2,3,4]. These challenges often lead to two key limitations in existing methods: insufficient capture of global contextual features and inadequate extraction of discriminative multi-scale representations [5,6].

With the advancement of deep learning, a variety of detection models have been applied to this domain. Early research primarily focused on two-stage detectors. Yang et al., for instance, employed Faster R-CNN [7] to localize pigs and their heads for recognizing feeding behavior [8]. Tu et al. later introduced PigMS R-CNN, integrating ResNet-101 with a Feature Pyramid Network (FPN) and soft-NMS to mitigate false detections in dense scenarios [9]. However, their reliance on region proposal generation leads to slow inference speeds, limiting their practicality for real-time monitoring. This computational bottleneck has driven a shift in research focus toward more efficient single-stage detectors [10].

For instance, Shen et al. demonstrated that YOLOv3 achieved comparable accuracy to Faster R-CNN in piglet detection while being 1.5 times faster, highlighting the superior speed–accuracy trade-off of single-stage detectors [11]. Subsequent research has largely focused on improving YOLO-series architectures for pig detection [12], incorporating attention mechanisms [13,14,15,16], advanced feature pyramids [14,17], and other specialized modules to enhance performance in complex scenarios.

The evolution of single-stage detectors has continued with the recent introduction of the Real-Time Detection Transformer (RT-DETR) [18]. This end-to-end model leverages the transformer architecture [19] to directly predict bounding boxes without anchors or complex post-processing, offering promising performance and real-time capability. Therefore, we adopt RT-DETR as our baseline. Nonetheless, when applied to the specific challenges of pig detection, such as distinguishing individuals in dense groups under complex conditions, we identify two potential limitations in its default architecture: its capacity for global feature capture and the richness of its multi-scale feature extraction could be further enhanced.

The core research gap lies in the lack of a unified model that effectively integrates long-range global semantics with hierarchical multi-scale local details. While some methods excel in local feature extraction, they often fail to model the broader contextual relationships necessary for disambiguating occluded or overlapping pigs. Conversely, global modeling approaches may overlook fine-grained details critical for precise localization under scale variations. This gap becomes especially pronounced in densely populated and visually cluttered farming environments [20,21].

To address these specific limitations, we propose VM-RTDETR, a novel detection model that synergistically enhances the RT-DETR framework. The main contributions of this work are summarized as follows:

- We introduce a Vision State-Space Duality (VSSD) module into the backbone network. Its core, a novel Non-Causal State-Space Duality (NC-SSD) mechanism, overcomes the unidirectional constraint of traditional state-space models by enabling bidirectional contextual modeling. This allows for highly efficient parallel computation while significantly strengthening the model’s ability to capture global structural features and long-range dependencies within an image.
- We design a Multi-Scale Efficient Hybrid Encoder (M-Encoder). This module utilizes parallel multi-scale depth-wise convolutional kernels to perform hierarchical feature extraction, simultaneously capturing fine-grained local details and broader contour information. This design effectively enriches the feature representation, improving the model’s robustness to scale variations caused by differing viewing distances and individual pig sizes.
- Through extensive experiments, we demonstrate that our VM-RTDETR model achieves state-of-the-art performance on challenging pig detection datasets, significantly boosting key metrics such as AP, $AP_{50}$, and $AP_{75}$ compared to existing mainstream approaches. The model provides a robust and efficient solution for intelligent livestock farming.

## 2. Materials and Methods

### 2.1. Dataset

**Figure 1:**
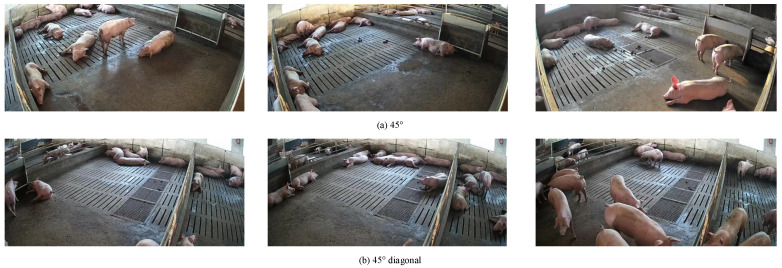
Collected data samples for pigs.

The dataset utilized in this study was collected from a large-scale commercial pig farm located in Linfen City, Shanxi Province. The pig house features an enclosed structure where pigs at different growth stages are physically separated by railings. The flooring is constructed with concrete and textured cement panels, providing both ventilation and liquid drainage capabilities. Video data were acquired using six Hikvision cameras (Hangzhou, China) strategically installed at various locations within the facility. All cameras were configured to record at a resolution of 1920 × 1080 pixels (1080p) and a frame rate of 25 fps. Each camera was mounted approximately 1.7 m above the ground, an optimal height and angle for obtaining comprehensive and clear footage of the pigs. Data collection was conducted over a two-month period from August to October 2022, yielding approximately 2 TB of raw video data. An obtained dataset sample is shown in Figure 1.

To mitigate the impact of low-quality data on model training, a structured processing pipeline was implemented. Initially, video segments affected by insufficient lighting or extreme weather conditions were manually filtered out. The specific criteria for exclusion were as follows: Insufficient lighting was defined as scenes where the overall illumination was too dark to distinguish the contours of pigs clearly without digital enhancement, typically corresponding to nighttime hours or periods of severe overcast. Extreme weather conditions primarily referred to heavy rain or snow that visibly obscured the camera lens or significantly degraded image clarity. Subsequently, frames were extracted at 20 s intervals from the validated videos to construct a preliminary image set in JPG format. Subsequently, a manual visual inspection was conducted to remove any remaining blurred or low-quality images, resulting in a refined dataset of 8070 unique, high-quality original images for model training and validation. This set of 8070 original images constitutes our final curated dataset before any augmentation.

**Figure 2:**
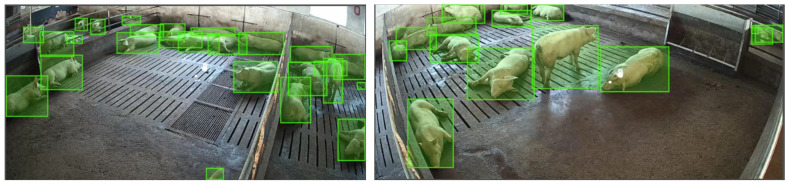
Some examples of annotated images.

The selected images were annotated using the Make Sense.ai online annotation tool (https://www.makesense.ai/). The annotation process was conducted by two annotators. To ensure consistency and accuracy, all annotations were cross-validated, meaning that each annotation created by one annotator was reviewed and verified by the other. As this study focuses solely on pig detection, a single class label, “pig”, was used for all bounding boxes. The bounding boxes were stored in the YOLO format, where each annotation in the TXT files contains the values <class_id>, <x_center>, <y_center>, <width>, and <height>, with all coordinates being normalized relative to the image dimensions. The annotations from the TXT files were subsequently converted into a structured JSON format for ease of use. The final JSON format follows the standard COCO dataset schema, containing keys for “images”, “annotations”, and “categories”. In this COCO-compliant JSON, the bounding box for each object is stored as a list [x_min, y_min, width, height], where the coordinates (x_min, y_min) are the absolute pixel values of the top-left corner of the bounding box, and width and height are the absolute dimensions in pixels. Some annotation examples are shown in Figure 2.

**Figure 3:**
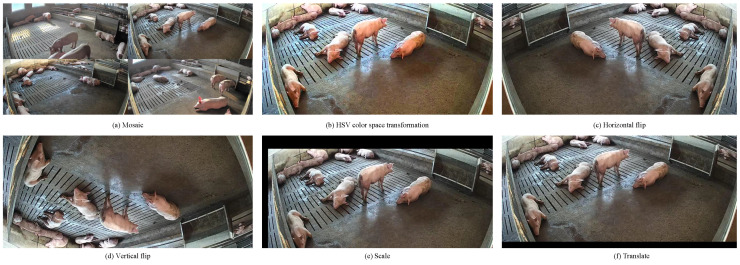
Some examples of data augmentation.

To enhance model performance and prevent overfitting, several data augmentation techniques were employed during the model training process. These included Mosaic (randomly combining four images into one), HSV color space enhancement (increasing saturation by 50% and brightness by 30%), horizontal flipping, vertical flipping, scaling, and translation (shifting images by 50 pixels along the *x*-axis and 100 pixels along the *y*-axis). Examples of augmented images are shown in Figure 3.

To ensure the generalization ability of the model and avoid bias introduced by random splitting, it was ensured that the training and test sets had similar distributions. Furthermore, to ensure the rigor of model validity evaluation and enhance the reproducibility of the experiments, the dataset of 8070 original images was randomly split with a fixed random seed to guarantee consistency in the partitioning. The value of the random seed used for this split was 42. This process resulted in a training set (6955 images) and a held-out test set (1115 images). No separate validation set was created. The test set was used exclusively for the final performance evaluation reported in this paper and was not used for model selection, early stopping, or hyperparameter tuning at any stage.

### 2.2. The Proposed Model, VM-RTDETR

**Figure 4:**
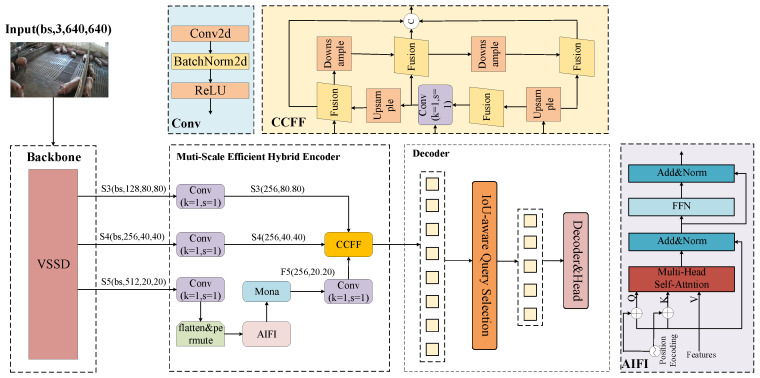
VM-RTDETR network structure diagram. The **AIFI** indicates Attention-based Intra-scale Feature Interaction module, the **CCFF** denotes the CNN-based Cross-scale Feature Fusion module, and the **Mona** indicates Multi-cognitive Visual Adapter module.

The overall architecture of the proposed pig detection model, VM-RTDETR, is illustrated in Figure 4. It consists of three main components: a feature extraction backbone, a Multi-Scale Efficient Hybrid Encoder (M-Encoder), and a decoder with a prediction head. The core innovations include the introduction of a VSSD [22] module into the feature extraction backbone and the novel design of the Multi-Scale Efficient Hybrid Encoder (M-Encoder). These enhancements effectively address the limitations of existing models in capturing global features and extracting rich multi-scale representations under complex farming environments.

#### 2.2.1. Backbone

**Figure 5:**
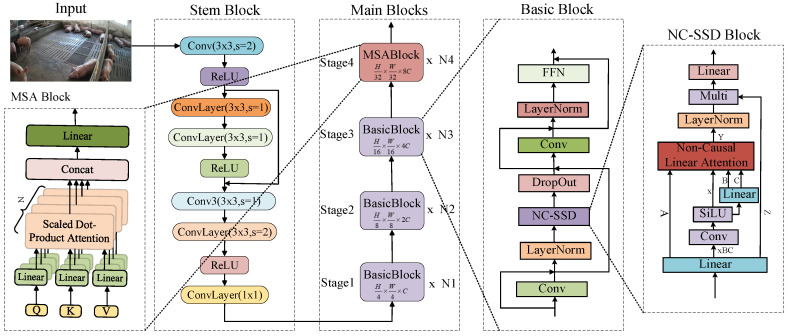
The feature extraction backbone network architecture of the VSSD module.

The RT-DETR model employs ResNet [23] as its backbone network, which exhibits certain limitations in pig detection tasks. Since ResNet’s convolutional operations primarily focus on local feature extraction, the resulting feature representations lack global contextual information, thereby compromising detection performance. Furthermore, during deep feature extraction, ResNet tends to lose high-frequency details, making precise localization challenging. Although RT-DETR [18] incorporates an Attention-based Intra-scale Feature Interaction (AIFI) module and CNN-based Cross-scale Feature Fusion (CCFF) module in its later stages, the initial feature representation inadequacy of the backbone hinders these modules from fully compensating for the lost information, ultimately affecting overall detection accuracy. To address these issues, the original ResNet backbone of the standard RT-DETR was replaced with our novel Vision State-Space Duality (VSSD) backbone in the proposed VM-RTDETR model. The structure of this new VSSD backbone is illustrated in Figure 5. This complete replacement is crucial, as the VSSD backbone is specifically designed to overcome the limitations of ResNet by effectively capturing global structural features with long-range dependencies in images through its core NC-SSD mechanism.

As illustrated in Figure 5, the VSSD module is designed to overcome the limitations of ResNet by incorporating a novel Non-Causal State-Space Duality (NC-SSD) mechanism as its core component. This mechanism breaks through the causal constraints of traditional state-space models (SSMs) and enables efficient modeling of global image features. Its core improvement lies in reformulating the state transition logic. By discarding magnitude information in the interaction between hidden states and inputs while preserving relative weights, it eliminates the limitation of causal masking on information flow.

In sequential models, causal masking ensures that the state at time step *t* only depends on past inputs, which is crucial for tasks like language modeling. This causal constraint is a fundamental characteristic of many traditional state-space models (SSMs), including the selective scan mechanism (SSM) that underpins recent vision models like VMamba [24]. However, this constraint is unnatural for images, where pixels are spatially correlated without an inherent temporal order. Applying such causal SSMs to image patches serialized into a 1D sequence thus unnecessarily restricts the model’s ability to integrate information from all parts of the image, as the representation at any position cannot be informed by subsequent tokens.

The core improvement of our proposed NC-SSD mechanism lies in its fundamental reformulation of the state transition logic to explicitly break this causal dependency. In contrast to the sequential, unidirectional processing of causal SSMs, the NC-SSD transforms the traditional recursive process into a global, non-causal accumulation operation (as formalized in Equation (4)). This key distinction allows every token in the sequence to independently and directly contribute to the hidden state of every other token, enabling truly bidirectional and parallel contextual modeling. This design is inherently more aligned with the physics of image data, where the understanding of a local feature can be refined by global contextual information from any other part of the image, leading to more effective global feature representation compared to the directionally constrained scans of prior SSM-based vision approaches.

In the traditional State-Space Duality (SSD) model [25], “SSD” refers specifically to State-Space Duality and should not be confused with the “Single Shot Detector” in object detection. The hidden state updates rely on historical input (causality), formulated as Equation (1):

(1) $$h\left(t\right)=A_{t}h\left(t-1\right)+B_{t}x\left(t\right),y\left(t\right)=C_{t}h\left(t\right)$$

where $A_{t}$ denotes a scalar transformation coefficient at time *t*, which functions to adjust the contribution weight of the current input feature to the hidden state. $B_{t}$ represents the input projection matrix at time *t*, which linearly transforms the current input feature x(t) into the dimension space of the hidden state. In an SSM, $A_{t}$ is used to control the retention ratio of historical hidden states; *h*(*t*−1) denotes the historical hidden state, carrying the knowledge and features accumulated from previous input information; and x(t) corresponds to the image token information input at the current moment, serving as the source of the latest information. This formula clearly shows that in traditional SSD, the current hidden state h(t) heavily depends on the previous hidden state *h*(*t*−1) and the current input x(t). Such a causal update mechanism limits the model’s ability to integrate global information efficiently. The matrix *C* is the output projection matrix, which transforms the high-dimensional hidden state h(t) into the model’s output y(t).

To overcome the causal constraints inherent in the traditional State-Space Duality (SSD), this study formally derives the Non-Causal State-Space Duality (NC-SSD) mechanism through a rigorous recursive expansion process, thereby revealing the global cumulative nature of the hidden state. Based on the hidden state update rule of traditional SSD, to deconstruct its temporal dependencies, this study performs a step-by-step expansion of the aforementioned recursive equation. By successively substituting h(t−1) and subsequent states, the following expansion is obtained:

(2) $$\begin{array}{cc} h(t) & =A_{t}h\left(t-1\right)+B_{t}x\left(t\right)\\ \; & =A_{t}\left[A_{t-1}h\left(t-2\right)+B_{t-1}x\left(t-1\right)\right]+B_{t}x\left(t\right)\\ \; & =A_{t}A_{t-1}h\left(t-2\right)+A_{t}B_{t-1}x\left(t-1\right)+B_{t}x\left(t\right)\\ \; & =A_{t}A_{t-1}\left[A_{t-2}h\left(t-3\right)+B_{t-2}x\left(t-2\right)\right]+A_{t}B_{t-1}x\left(t-1\right)+B_{t}x\left(t\right)\\ \; & =A_{t}A_{t-1}A_{t-2}h\left(t-3\right)+A_{t}A_{t-1}B_{t-2}x\left(t-2\right)+A_{t}B_{t-1}x\left(t-1\right)+B_{t}x\left(t\right)\\ \; & \;\;\vdots \\ \; & =\left(\prod_{j=k+1}^{t}A_{j}\right)h\left(k\right)+\sum_{i=k+1}^{t}\left(\prod_{j=i+1}^{t}A_{j}\right)B_{i}x\left(i\right),\;\mathrm{for}\;\mathrm{any}\;k<t \end{array}$$

When the recursive expansion reaches the initial state h(0) and setting h(0)=0, the above expansion simplifies to a pure accumulation form:

(3) $$h\left(t\right)=\sum_{i=1}^{t}\left(\prod_{j=i+1}^{t}A_{j}\right)B_{i}$$

In this expression, the term $\prod_{j=i+1}^{t}A_{j}$ represents the multiplicative accumulation effect of the scalar coefficients $A_{j}$ from time step i+1 to *t*. This derivation mathematically confirms that the hidden state h(t) at any time step *t* is essentially an explicit weighted sum of all historical inputs x(i) in the sequence. The weights are jointly determined by the model parameters $B_{i}$ and the sequential coefficients $A_{j}$.

Based on this mathematical essence, the NC-SSD mechanism further reconstructs the complex cross-time-step dependencies encapsulated by the product term $\prod_{j=i+1}^{t}A_{j}$ into an adaptive importance weight $\frac{1}{A_{i}}$ applied to the current input x(i). This reformulation embodies its core innovation: transforming the sequential, state-dependent update into a parallelizable, input-weighted aggregation.

Mathematically, this reformulation leads to an equivalent, global representation of the hidden state, as shown in Equation (4):

(4) $$h\left(t\right)=\sum_{i=1}^{t}\frac{1}{A_{i}}B_{i}x\left(i\right)=\sum_{i=1}^{t}\frac{1}{A_{i}}Z_{i},\;\mathrm{where}\;Z_{i}=B_{i}x\left(i\right).$$

Here, $\frac{1}{A_{i}}$ serves as a learnable importance weight that directly and independently governs the contribution intensity of the *i*-th token x(i) to the final state, thereby completely eliminating the causal constraints of the traditional model. $B_{i}$ represents the input projection weight (a scalar learnable parameter) of the *i*-th token, used to linearly scale the current input feature x(i), mapping it to a base value that directly influences the contribution to the hidden state. The tensor *Z* is introduced in the derivation of the Non-Causal SSD (NC-SSD) and represents the projected input token. It is defined as $Z_{i}=B_{i}x\left(i\right)$, meaning that each token x(i) is first transformed by the input-dependent projection matrix $B_{i}$.

The essence of the accumulation process $\sum_{i=1}^{t}\frac{1}{A_{i}}B_{i}x\left(i\right)$ is the weighted aggregation of all sequence features. In visual tasks, after an image is flattened into a 1D sequence, the correlation between adjacent pixels or regions may be disrupted due to serialization (for example, adjacent pixels in a 2D image might be far apart in the 1D sequence). NC-SSD enables the features of each pixel to directly participate in the construction of the final hidden state through global accumulation. For instance, the pixel feature x(1) at the edge of the image and the pixel feature x(t) at the center can directly contribute to h(t) through their respective weights of $\frac{1}{A_{1}}B_{1}$ and $\frac{1}{A_{t}}B_{t}$. This mechanism captures long-range spatial dependencies more efficiently without relying on the sequential transmission of intermediate token states.

#### 2.2.2. M-Encoder

**Figure 6:**
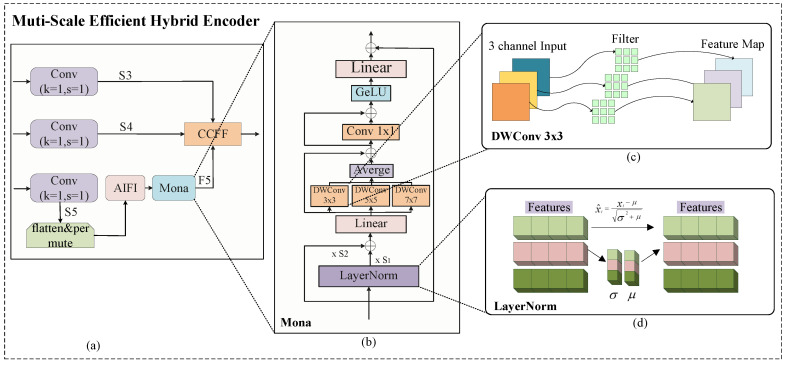
The architecture of the Multi-Scale Efficient Hybrid Encoder module M-Encoder. Here, (**a**) is the architecture of the Multi-Scale Efficient Hybrid Encoder M-Encoder we proposed, where the outputs of the three parallel depth-wise convolutional branches are fused using an element-wise average operation. (**b**) is the core module Mona [26] in the M-Encoder, used for multi-scale feature extraction. (**c**) is the detailed structure diagram of DWConv 3 × 3 . (**d**) is the detailed part of LayerNorm.

The S3, S4, and S5 features extracted by VM-RTDETR in the backbone network module were fed into the improved Multi-Scale Efficient Hybrid Encoder (M-Encoder). Compared to the shallower S3 and S4 features, the S5 feature contains deeper, higher-level, and semantically richer information. The newly designed M-Encoder employs parallel multi-scale depth-wise convolutional filters with kernel sizes of 3 × 3, 5 × 5, and 7 × 7. This multi-scale design is grounded in the cognitive principle that visual information is processed at varying receptive fields to capture both local details and global context. It effectively overcomes the limitations of traditional linear adapters in visual signal processing and breaks through the constraint of single-scale feature extraction. The selected kernel sizes effectively balance feature richness and parameter efficiency. The 3 × 3 kernels capture fine-grained local features (e.g., textures and edges of pigs), the 5 × 5 kernels extract intermediate structural patterns (e.g., limbs and head contours), and the 7 × 7 kernels integrate broader contextual information (e.g., overall body posture and spatial relationships among multiple pigs). This configuration enables a comprehensive representation of porcine features across varying scales, thereby significantly enhancing the model’s robustness in complex environments. The overall structure of the M-Encoder is shown in Figure 6.

The M-Encoder addresses the limitations of traditional single-scale encoding by incorporating parallel multi-scale convolutions, feature normalization, and cross-scale fusion. It accurately captures multi-dimensional visual patterns, providing more discriminative feature representations for pig detection.

The module first applies a hybrid normalization operation to the input feature $X_{0}$, comprising Layer Normalization (LN) and learnable scaling factors. The definition is as follows:

(5) $$X_{\mathrm{norm}}=s_{1}\cdot \mathrm{LN}\left(X_{0}\right)+s_{2}\cdot X_{0}$$

LN is applied to the input feature $X_{0}$ along the channel dimension, bringing the distribution of each channel closer to a standard normal distribution, and enhancing the learning consistency of the model across different channels. Here, $s_{1}$ and $s_{2}$ are learnable scalar parameters that are shared across all channels and adaptively adjust the global fusion weights between the LN features and the original feature $X_{0}$ during training. This design mitigates the loss of detail commonly associated with conventional normalization methods, allowing the model to flexibly balance stabilizing feature distributions and preserving original information. In the context of pig detection, this mechanism not only regularizes the feature distribution to facilitate model optimization but also preserves critical details such as the original appearance and texture of the pigs.

The normalized feature $X_{\mathrm{norm}}$ is then processed through three parallel depth-wise convolution (DWConv) branches with kernel sizes of 3 × 3, 5 × 5, and 7 × 7. This process is defined as

(6) $$X_{\mathrm{out}}=\mathrm{Avg}\left({\mathrm{DWConv}}_{3\times 3}\left(X_{\mathrm{norm}}\right),{\mathrm{DWConv}}_{5\times 5}\left(X_{\mathrm{norm}}\right),{\mathrm{DWConv}}_{7\times 7}\left(X_{\mathrm{norm}}\right)\right)+X_{\mathrm{norm}}$$

Here, ${DWConv}_{k}$ denotes a depth-wise convolution with a kernel size of *k*. This operation first performs spatial convolution channel-wise on $X_{\mathrm{norm}}$ to capture within-channel spatial patterns (such as local textures and edges of pigs), followed by point-wise convolution (1 × 1 convolution) to fuse information across channels. The Avg operation performs an element-wise averaging of the three multi-scale feature maps to integrate their multi-scale features, enabling the collaborative combination of detailed features from 3 × 3 convolution, medium-scale structural features from 5 × 5 convolution, and global contextual features from 7 × 7 convolution. To clearly illustrate the internal structure, a detailed schematic of the DWConv 3 × 3 operation is provided in Figure 6c. The architectural principles for the DWConv 5 × 5 and DWConv 7 × 7 branches are analogous to those of the DWConv 3 × 3 branch, differing only in the kernel size of the depth-wise convolution operation. Therefore, separate sub-figures for these similar structures are omitted for conciseness. The residual connection helps alleviate gradient vanishing and ensures effective propagation of multi-scale features to subsequent processing stages.

To meet the input requirements of the downstream detection, the multi-scale features are dimensionally expanded and fused with the original information. The mathematical expression is as follows:

(7) $$Y=X_{0}+UpProjection\left(\mathrm{GeLU}\left({\mathrm{Conv}}_{1\times 1}\left(f_{\mathrm{dw}}\right)\right)\right)$$

The 1 × 1 convolution operation enables preliminary integration of multi-scale features along the channel dimension. The UpProjection operation is defined as a linear (fully connected) layer that restores the channel dimension of the features. Specifically, it projects the feature map processed by the 1 × 1 convolution and GeLU activation back to the original channel depth of $X_{0}$, ensuring dimensional consistency for the subsequent residual addition.

By incorporating a residual connection from $X_{0}$, essential details in the original input, such as distinctive color patterns and limb structures of pigs, are preserved, preventing the loss of critical information during multi-scale encoding. This design allows the original and multi-scale enhanced features to complement each other, significantly improving the richness and completeness of feature representation.

Finally, the Mona module performs cross-scale feature fusion to effectively integrate multi-source information, combining its processed features with other scale features from the backbone network. This leverages multi-scale information in the image comprehensively. Specifically, the S3 and S4 features are first pre-processed; a dedicated 1 × 1 convolution (without weight sharing between scales) is applied to each to unify their channel dimensions to match those of the S5 feature map processed by the Mona module. This is followed by an upsampling operation, implemented using bilinear interpolation, to align their spatial sizes with the S5 feature map. Finally, the processed S3 and S4 feature maps are element-wise summed with $F_{S5}^{Mona}$ to obtain the final multi-scale feature fusion result $F_{\mathrm{final}}$. The mathematical expression can be described as follows:

(8) $$F_{\mathrm{final}}=F_{S5}^{\mathrm{Mona}}+\mathrm{Upsample}\left(F_{S4}\right)+\mathrm{Upsample}\left(F_{S3}\right)$$

Here, $F_{S5}^{\mathrm{Mona}}$ denotes the S5 feature processed by the Mona module, and Upsample refers to the upsampling operation that adjusts the spatial size of feature maps through interpolation or other methods to achieve dimensional alignment.

#### 2.2.3. Decoder

The configuration of the decoder, including the number of queries, the parameters of the IoU-aware query selection mechanism, and the absence of NMS (as it is an end-to-end detector), is identical to that of the original RT-DETR [18]. For specific values and implementation details, we direct the reader to the aforementioned reference, as these are core components of the baseline model which we utilized without modification.

#### 2.2.4. Synergistic Design of VSSD and M-Encoder

The architectural innovation of VM-RTDETR extends beyond the independent enhancements of the VSSD backbone and the M-Encoder. The most significant contribution lies in their synergistic collaboration, which was deliberately designed to address the two intertwined fundamental challenges in pig detection: modeling long-range contextual dependencies and capturing discriminative features across multiple scales.

As illustrated in Figure 4, the VSSD backbone and the M-Encoder form two complementary feature processing pathways:

The VSSD backbone, empowered by the NC-SSD mechanism, establishes a global contextual understanding of the entire image. By breaking through the causal constraints of traditional SSMs, it allows any pixel or region in the image to directly influence the representation of any other, regardless of distance. This capability is crucial for reasoning in complex scenarios, such as inferring the presence of an occluded pig based on the overall herd structure or understanding spatial relationships between distant individuals.

The M-Encoder operates as a powerful local multi-scale feature extractor. Its parallel convolutional kernels with varying receptive fields (3 × 3, 5 × 5, 7 × 7) are specialized in capturing hierarchical visual patterns. The 3 × 3 kernels focus on fine-grained textures, the 5 × 5 kernels aggregate these into intermediate contours, and the 7 × 7 kernels integrate broader contextual shapes.

The synergy emerges from the fusion of these pathways’ outputs. The VSSD pathway provides a “top-down” semantic guide, informing the model about the global scene layout and the likely locations and relationships of pig entities. The M-Encoder pathway provides “bottom-up”, detailed, multi-scale evidence about local appearances and boundaries. The subsequent feature fusion, particularly in the CCFF module and the decoder, integrates these two streams of information.

This synergistic design ensures that the final feature representation used for detection is both semantically coherent and locally precise. For instance, the global context from VSSD can help resolve ambiguities in crowded regions, while the multi-scale details from the M-Encoder enable precise localization of individual pigs, even when their sizes vary greatly or they are partially hidden. This collaborative mechanism is the cornerstone of VM-RTDETR’s robustness and superior performance, effectively tackling the core limitations of prior methods that often excelled in only one of these two aspects.

### 2.3. Evaluation Metric

To comprehensively evaluate the performance of our pig detection model, we adopt several standard metrics in object detection. The primary metrics are based on Average Precision (AP) at different Intersection-over-Union (IoU) thresholds. The IoU measures the overlap between a predicted bounding box and the ground truth.

Specifically, we report the following:

- $AP_{50:95}$ (abbreviated as AP): The mean AP averaged over IoU thresholds from 0.5 to 0.95 with a step size of 0.05. This is the primary metric for comprehensive performance evaluation.
- $AP_{50}$: The AP at an IoU threshold of 0.5.
- $AP_{75}$: The AP at a stricter IoU threshold of 0.75.

The AP for a single class is defined as the area under the precision–recall curve, as shown in Equation (9):

(9) $$AP=\int_{0}^{1}P\left(R\right)dR$$

where P(R) denotes precision as a function of recall. Accordingly, the mAP for multiple categories is defined by Equation (10):

(10) $$mAP=\frac{1}{N}\sum_{n=1}^{N}AP_{n}$$

where *N* is the number of categories and $AP_{n}$ is the AP for the *n*-th category.

Since our dataset contains only one class (pig), the mean Average Precision (mAP) across all classes is equivalent to the AP for the pig class.

To analyze the model’s performance across objects of different sizes, we also report

- $AP_{m}$: AP for medium objects (32×32≤ pixel area < 96 × 96);
- $AP_{l}$: AP for large objects (pixel area ≥96×96).

It is noteworthy that the standard metric for small objects ($AP_{s}$, area < 32 × 32 pixels) is not applicable to our dataset. The distribution of bounding box areas confirmed the absence of a significant population of “small” objects as per the COCO standard, as the pigs in our images, even at a young age, predominantly fall into the medium or large size categories due to the fixed camera perspective and resolution.

Besides detection accuracy, we evaluate the model’s efficiency utilizing two metrics:

- Params: The total number of trainable parameters, indicating the model’s size.
- GFLOPs (Giga Floating-Point Operations): The total number of floating-point operations required for a single forward pass, measured in billions. It reflects the model’s computational complexity. A lower value indicates higher computational efficiency.

## 3. Experimental Results and Analyses

To thoroughly evaluate the effectiveness of the proposed VM-RTDETR model for pig detection, we conducted a series of comprehensive experiments. The following sections detail the experimental setup, comparative results with mainstream models, and an in-depth analysis of the findings.

### 3.1. Experimental Setup

The experiments were conducted on a server running Ubuntu 18.04, equipped with eight NVIDIA Tesla P100 GPUs (each with 16GB memory). We utilized the PyTorch 2.1.1 deep learning framework with CUDA 11.8 and Python 3.10.17. The input image size was set to 640 × 640, with a batch size of 4 for 100 training epochs. The Adam optimizer was adopted with an initial learning rate of 0.0001, a weight decay of 0.0001, and beta parameters ($\beta_{1}$, $\beta_{2}$) set to (0.9, 0.999). A standard random sampler was used for constructing training batches. Detailed hardware and software configurations are listed in Table 1.

### 3.2. Comparison of Different Models

To validate the effectiveness of VM-RTDETR, we compared it against current mainstream detectors, including Faster R-CNN [7], YOLOv5s/n, YOLOv6s/n, YOLOv7 [27], YOLOv8s/n, YOLOv10n, YOLOv12s/n, and R50-RTDETR [18]. The quantitative results are summarized in Table 2.

As shown in Table 2, VM-RTDETR achieves state-of-the-art performance across core metrics. It attains an AP of 60.9%, significantly outperforming all compared models. We highlight the following key comparisons: Our model surpasses the widely used YOLOv7 by a relative improvement of 1.67% in AP and, more importantly, exceeds its baseline R50-RTDETR by a relative improvement of 2.35%. Similar leading trends are observed in $AP_{50}$ (95.5%) and $AP_{75}$ (63.3%), confirming comprehensive improvements in both overall detection quality and precise localization accuracy. Significantly, VM-RTDETR achieves these superior results with enhanced efficiency, requiring only 97.9 GFLOPs and 28.4 M parameters, a significant reduction compared to R50-RTDETR (137.7 GFLOPs, 42.7 M parameters). Additionally, a single dash (-) indicates that the metric was not reported in the evaluation output for that specific model ($AP_{75}$ for YOLOv7). A double dash (–) indicates that the metrics were not available or not computed due to architectural incomparability with the standard evaluation framework.

**Figure 7:**
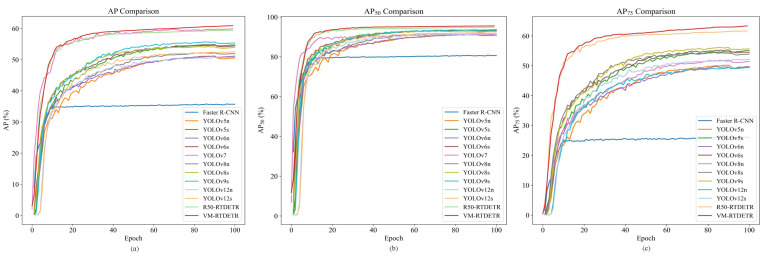
Performance comparison of AP curves of different models.

The convergence behavior and performance stability of different models during training are compared in Figure 7.

As shown in Figure 7a, the AP curve of VM-RTDETR not only converges to the highest value but also exhibits a faster and more stable rise compared to other models. Similarly, its $AP_{50}$ curve in Figure 7b maintains a clear leading advantage, indicating robust localization capability from the early stages. Most notably, the $AP_{75}$ curve in Figure 7c shows a substantial performance gap, especially in later epochs, underscoring the model’s enhanced capability for high-precision bounding box regression under stringent IoU thresholds.

**Figure 8:**
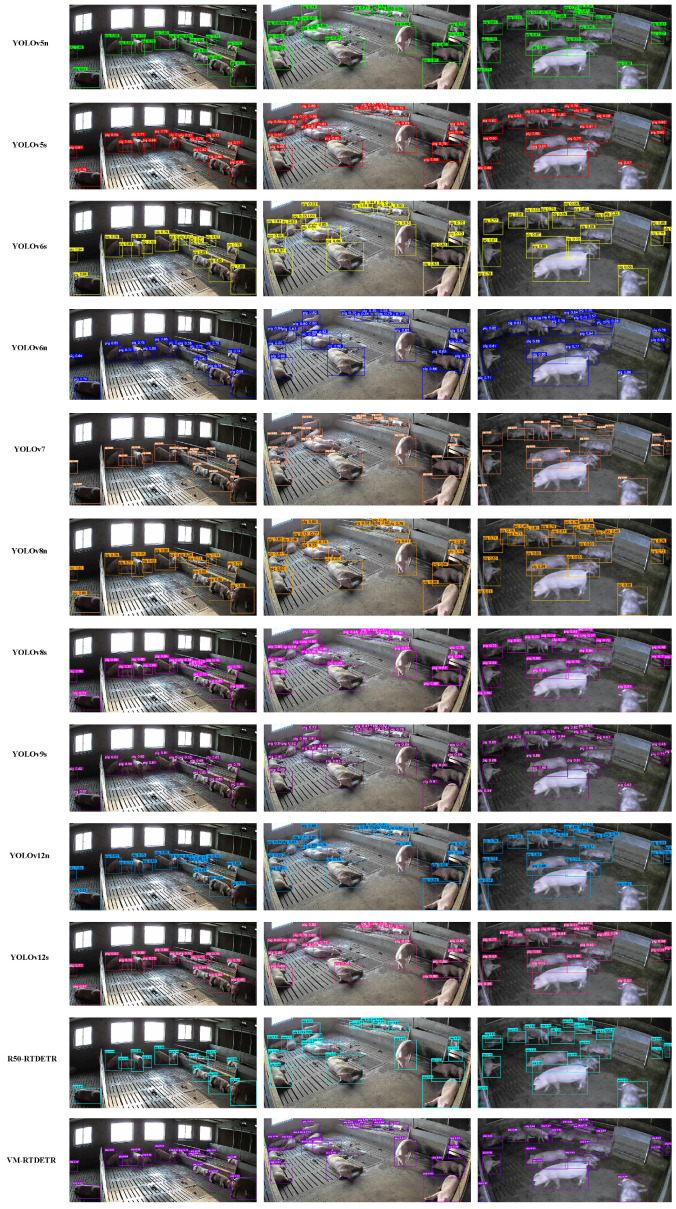
Comparison of detection boxes and accuracy of different models.

We further compare the bounding box quality and detection accuracy qualitatively in Figure 8. The results demonstrate that VM-RTDETR produces the most precise and complete bounding boxes, indicating a superior ability to localize pig targets and delineate their boundaries accurately in complex scenes.

The superior performance of VM-RTDETR can be attributed to its synergistic architectural design. First, the VSSD backbone, empowered by the NC-SSD mechanism, breaks through the causal constraints of traditional state-space models. This enables efficient modeling of long-range dependencies and significantly enhances the extraction of global structural features from the image. Second, the M-Encoder tackles the challenge of scale variation by replacing single-scale processing with a parallel architecture employing 3 × 3, 5 × 5, and 7 × 7 convolutional kernels. This design simultaneously captures fine-grained details (via 3 × 3 kernels), intermediate contours (via 5 × 5 kernels), and broader spatial contexts (via 7 × 7 kernels). The subsequent fusion of these multi-scale features constructs a comprehensive hierarchical representation. Consequently, through the synergistic optimization of the VSSD backbone and M-Encoder, VM-RTDETR demonstrates stronger robustness and higher detection accuracy in complex scenarios (such as occlusion and lighting variations), providing an efficient and precise solution for automated pig detection.

### 3.3. Ablation Studies

#### 3.3.1. Verification of the VSSD Module

For the sake of simplicity in writing, we will represent the backbone as B and the encoder as E, represent the encoder as a transformer with T, and represent the M-Encoder as ME. To validate the superiority of the VSSD module, we integrated it into the RT-DETR framework and compared it against several established backbones, including ResNet-18, ResNet-34, ResNet-50, ResNet-101, and VMamba [24]. The results are summarized in Table 3.

As shown in Table 3, the V-RTDETR model achieves the best overall performance, demonstrating comprehensive advantages across all core metrics. Specifically, it attains an AP of 60.4%, achieving a relative improvement over R18-RTDETR, R34-RTDETR, R50-RTDETR, R101-RTDETR, and VMamba-RTDETR of 1.51%, 1.34%, 1.51%, 1.17%, and 0.33%, respectively. Similarly, its $AP_{50}$ of 95.3% is 0.42%, 0.11%, 0.42%, and 0.21% higher than those of the R18-RTDETR, R34-RTDETR, R50-RTDETR, and VMamba-RTDETR, respectively, comparable to that of the R101-RTDETR. The advantage is even more pronounced on the stricter $AP_{75}$ metric (62.9%), which shows relative improvements of 1.94%, 1.78%, 2.11%, 1.29%, and 0.64% over the comparison models. Notably, the V-RTDETR achieves an excellent balance between computational complexity and parameter count. It is important to highlight that the VSSD backbone alone brings a substantial reduction in computational cost, reducing GFLOPs by 28.9% (from 137.7 G to 97.9 G) compared to the R50-RTDETR baseline. While its GFLOPs (97.9 G) and parameters (28.4 M) are slightly higher than those of VMamba-RTDETR (87.0 G, 26.3 M) and R18-RTDETR (61.1 G, 20.0 M), they are substantially lower than R50-RTDETR (137.7 G, 42.7 M) and R101-RTDETR (260.6 G, 76.4 M), while delivering comprehensively superior performance. Therefore, V-RTDETR achieves the optimal trade-off between accuracy and efficiency.

**Figure 9:**
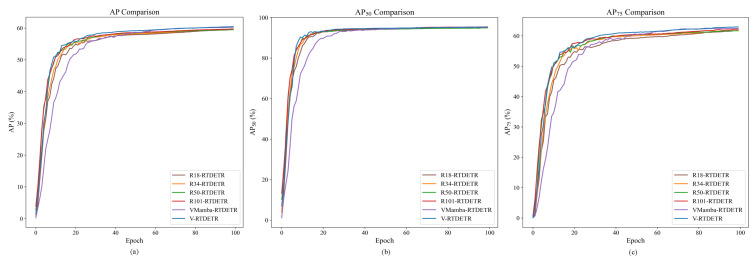
Comparison curves of AP values between R18-RTDETR, R34-RTDETR, R50-RTDETR, R101-RTDETR, VMamba-RTDETR, and V-RTDETR models.

**Figure 10:**
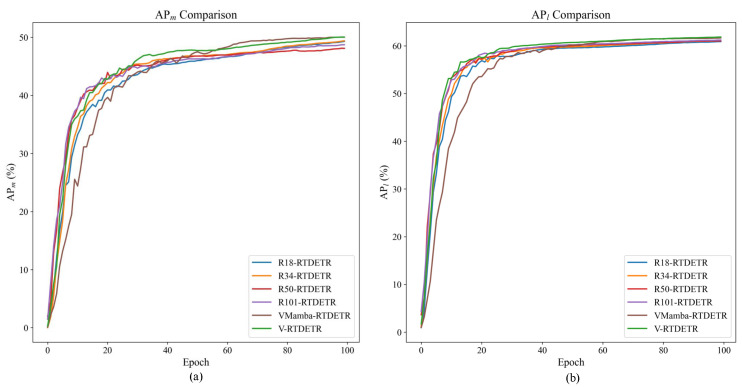
Comparison curves of $AP_{m}$ and $AP_{l}$ values between R18-RTDETR, R34-RTDETR, R50-RTDETR, R101-RTDETR, VMamba-RTDETR, and V-RTDETR models.

The convergence advantages of the VSSD backbone are visualized in Figure 9 and Figure 10, which plot the evaluation metrics against training iterations. Figure 9 shows that the curves for V-RTDETR for AP, $AP_{50}$, and $AP_{75}$ consistently dominate those of all baseline models throughout the training process, while Figure 10 further confirms that its curves for $AP_{m}$ and $AP_{l}$ also maintain a steady leading position compared to the same set of baselines. This demonstrates that our model not only achieves a higher final accuracy but also maintains a stable and leading performance advantage from the early stages of training.

**Figure 11:**
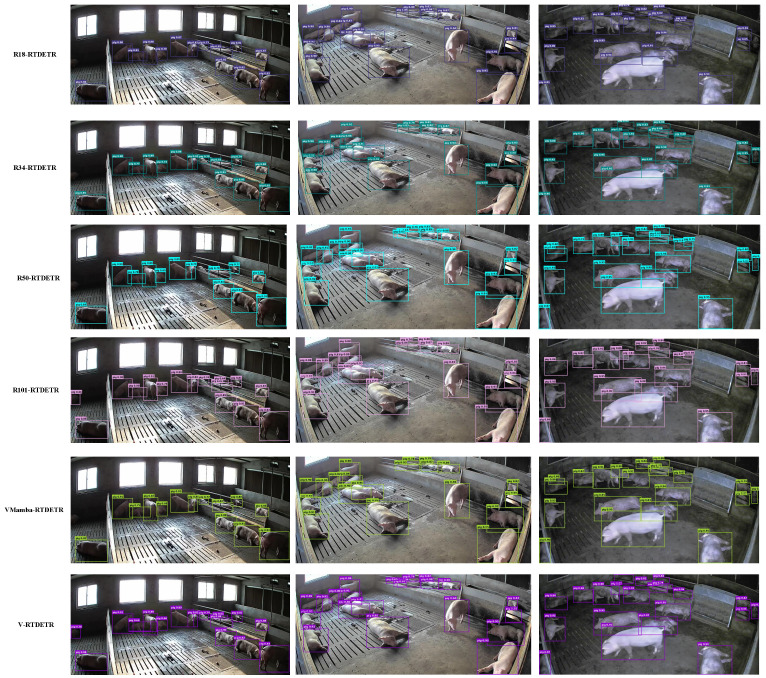
Comparison of the detection boxes and accuracy between R18-RTDETR, R34-RTDETR, R50-RTDETR, R101-RTDETR, VMamba-RTDETR, and V-RTDETR models.

Qualitative results are provided in Figure 11. V-RTDETR generates more precise bounding boxes and exhibits stronger robustness in challenging scenarios, such as occlusion, compared to the other backbone variants.

The superior performance of the model with the VSSD backbone can be attributed to the following reason: As the feature extraction backbone, the VSSD module adopts the NC-SSD mechanism. It breaks through the limitation of traditional SSMs, where hidden states only depend on historical inputs, and constructs a unified global hidden state. This enables each token to directly access complete contextual information, thus allowing the model to effectively capture global structural features with long-range dependencies in images.

#### 3.3.2. Verification of the M-Encoder Module

To assess the effectiveness of the proposed M-Encoder module, we integrate it into the RT-DETR encoder, forming the M-RTDETR model. A comparative analysis between the original RTDETR and M-RTDETR is presented in Table 4.

As shown in Table 4, the M-RTDETR model outperforms the baseline RTDETR across all evaluation metrics. Specifically, it achieves an AP of 59.8% (a gain of +0.50%), an $AP_{50}$ of 95.1% (+0.21%), an $AP_{75}$ of 62.3% (+1.14%), an $AP_{m}$ of 48.2% (+0.42%), and an $AP_{l}$ of 61.4% (+0.66%). These consistent improvements validate the contribution of the M-Encoder module. Importantly, these performance gains are achieved without increasing computational costs, as both models maintain identical GFLOPs (137.7 G) and parameters (42.7 M), demonstrating the efficiency of the M-Encoder design.

**Figure 12:**
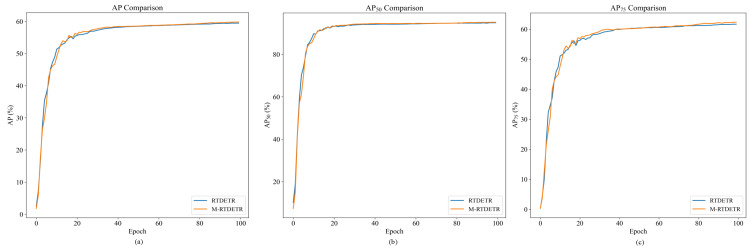
Comparison of AP values between RTDETR and M-RTDETR models.

**Figure 13:**
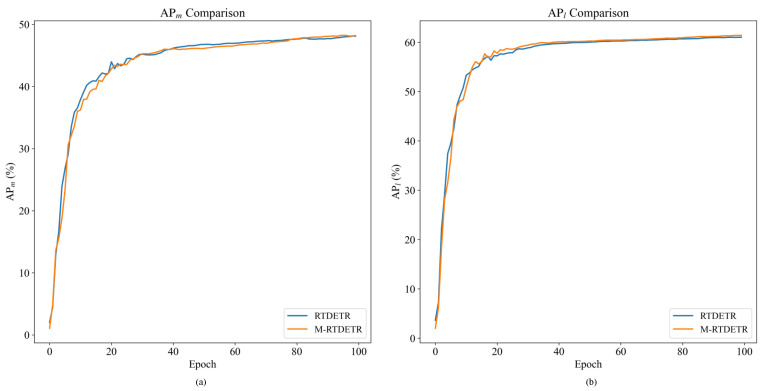
Comparison curves of $AP_{m}$ and $AP_{l}$ values between RTDETR and M-RTDETR models.

The training dynamics of M-RTDETR are further visualized in Figure 12 and Figure 13. Figure 12 shows that the convergence curves for AP, $AP_{50}$, and $AP_{75}$ of M-RTDETR consistently lie above those of RT-DETR across training epochs. This advantage is also evident in the medium- and large-object detection metrics ($AP_{m}$ and $AP_{l}$), as shown in Figure 13. The observed improvements for both medium and large objects underscore that the multi-scale fusion capability of the M-Encoder effectively handles pigs at various sizes and distances, which is a key requirement for robust herd monitoring in practical farming environments. Together, these figures demonstrate that the performance gain from the M-Encoder module is consistent and stable throughout the entire training process.

The performance contribution of the M-Encoder module is mainly reflected in its ability to extract parallel multi-scale features. By synchronously using convolution kernels of different scales such as 3 × 3, 5 × 5, and 7 × 7, this module constructs a hierarchical feature extraction system. Specifically, the 3 × 3 convolution kernel focuses on capturing local fine-grained features, the 5 × 5 convolution kernel is responsible for extracting medium-range contour information, and the 7 × 7 convolution kernel covers a broader spatial context. The parallel extraction and deep fusion of these multi-scale features enable the model to effectively deal with complex scenarios such as inconsistent target scales and mutual occlusion, thereby significantly improving the accuracy of recognition and localization for targets.

#### 3.3.3. Verification of the VM-RTDETR Model

To validate the effectiveness of our proposed modules, we design a series of ablation baselines: RTDETR (original architecture), V-RTDETR (with our VSSD backbone only), M-RTDETR (with our M-Encoder module only), and our full model, VM-RTDETR (integrating both the VSSD backbone and the M-Encoder module). This progressive comparison allows us to isolate the contribution of each component. The results, presented in Table 5, conclusively demonstrate the superiority of our complete VM-RTDETR model.

As shown in Table 5, our VM-RTDETR model achieves state-of-the-art performance across all metrics, demonstrating clear advantages over the baseline models (RTDETR, V-RTDETR, and M-RTDETR). Specifically, VM-RTDETR attains an AP of 60.9%, which is 2.35%, 0.83%, and 1.84% higher than RTDETR, V-RTDETR, and M-RTDETR, respectively. The performance gap is even more pronounced on more stringent metrics: a 63.3% $AP_{75}$ (representing a relative lead of 2.76%, 0.64%, and 1.61%) and a 50.7% $AP_{m}$ (representing a relative gain of 5.63%, 1.40%, and 5.19%). Consistent relative improvements are also observed in $AP_{50}$ (95.5%, +0.63%/+0.21%/+0.42%) and $AP_{l}$ (62.3%, +2.13%/+0.81%/+1.47%). Remarkably, VM-RTDETR achieves these performance gains with only 97.9 GFLOPs and 28.4 M parameters, substantially more efficient than both RTDETR and M-RTDETR (137.7 GFLOPs, 42.7 M parameters) while matching the efficiency of V-RTDETR, demonstrating an optimal balance between accuracy and computational cost.

**Figure 14:**
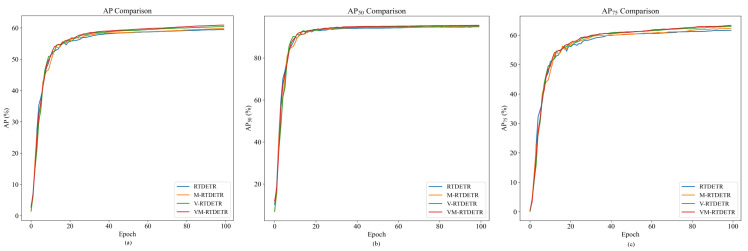
Comparison of AP values between RTDETR, M-RTDETR, V-RTDETR, and VM-RTDETR models.

**Figure 15:**
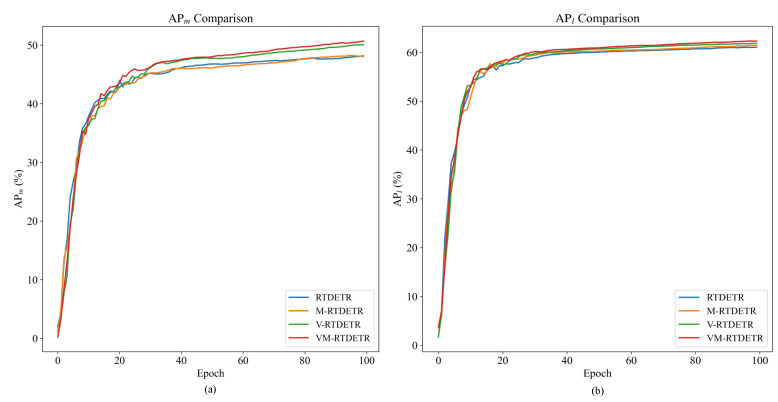
Comparison curves of $AP_{m}$ and $AP_{l}$ values between RTDETR, M-RTDETR, V-RTDETR, and VM-RTDETR models.

The performance advantages of our proposed VM-RTDETR are further visualized in Figure 14 and Figure 15, which plot the evaluation metrics against training iterations. As shown, the curves for VM-RTDETR (across AP, $AP_{50}$, $AP_{75}$, $AP_{m}$, and $AP_{l}$) consistently dominate those of the baseline models (RTDETR, V-RTDETR, and M-RTDETR) throughout the training process. This demonstrates that our model not only achieves superior final performance but also maintains a stable and leading convergence advantage from start to finish.

**Figure 16:**
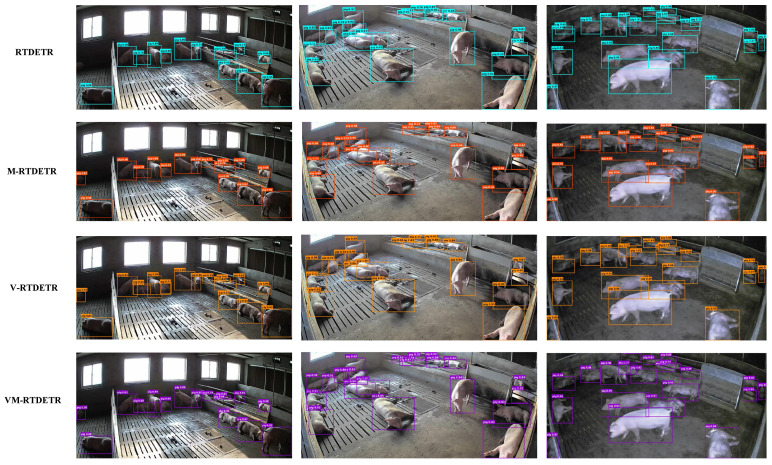
Comparison of detection boxes and accuracy of RTDETR, M-RTDETR, V-RTDETR, and VM-RTDETR models.

Figure 16 compares the detection boxes and detection accuracy of four models, RTDETR-R50, M-RTDETR, RTDETR-VSSD, and VM-RTDETR. As clearly shown in the first column of images, VM-RTDETR successfully detected pigs in the upper-left area that were missed by RTDETR-R50, while achieving higher detection accuracy compared to other models. In the second column, the original model failed to detect the seven overlapping pigs at the top, whereas VM-RTDETR accurately identified all pigs under occlusion. In the third column, VM-RTDETR detected the top pig under challenging lighting conditions, while other models failed, demonstrating superior detection accuracy and significantly reducing the missed detection rate. These comparative results visually corroborate the quantitative performance gains reported in Table 2 and Table 5, demonstrating VM-RTDETR’s practical advantages in handling real-world complexities such as occlusion, dense grouping, and lighting variations.

The superior performance of the VM-RTDETR model stems not only from the independent enhancements of its core components but, more critically, from their effective synergy. Specifically, the VSSD backbone establishes a comprehensive global contextual understanding of the image, while the M-Encoder module captures fine-grained local details through its multi-scale convolutional architecture. The subsequent fusion of these complementary representations, integrating high-level semantics with low-level spatial details, enables a more discriminative and robust feature representation. This synergistic design empowers the model to maintain high accuracy under challenging conditions, including illumination variations, significant scale differences, orientational diversity, and partial occlusions.

## 4. Discussion

This study proposes VM-RTDETR, a model that achieves significant performance gains in pig detection by synergistically enhancing global context modeling and multi-scale local feature extraction. The ablation studies confirm that the VSSD backbone and M-Encoder module are mutually reinforcing. The comparative analysis further elucidates the source of VM-RTDETR’s advantages. When compared to convolution-heavy architectures like the YOLO series [12,27], our model exhibits superior capability in capturing global contextual information, thanks to the VSSD module. Conversely, against the original RTDETR baseline [18], VM-RTDETR demonstrates a significantly enhanced capacity for multi-scale feature extraction due to the M-Encoder. This dual optimization of both global and local feature pathways distinguishes our approach and underpins its state-of-the-art performance.

Notwithstanding these promising results, we acknowledge certain limitations that provide valuable directions for future research. Firstly, the current study is constrained by its dataset, which was collected from a single commercial indoor farm in Shanxi Province using a fixed overhead-ish camera angle and primarily consists of grower-finisher pigs. This limits the model’s validated performance for other production systems (e.g., outdoor), geographical locations, camera viewpoints, or distinct animal classes like piglets. Secondly, while VM-RTDETR demonstrates excellent performance on pig detection, its scalability to multi-category livestock detection remains to be verified. The model’s generalization capability across species with diverse morphological characteristics (e.g., cattle, sheep, or poultry) requires further investigation through cross-species validation. Thirdly, although our model shows improved computational efficiency compared to the baseline R50-RTDETR, its practical deployment on resource-constrained edge devices commonly used in farm environments warrants additional optimization. Future work should explore model compression techniques and specialized optimization for edge deployment. Fourthly, while the current model exhibits robustness to environmental variations within our dataset, its performance under more extreme conditions—such as severe weather, drastically different farm architectures, or highly challenging lighting situations—needs further validation. To address these limitations, future work will prioritize expanding the dataset’s diversity in terms of farm types, locations, viewpoints, and animal categories. Furthermore, investigating multi-modal sensing approaches, particularly the fusion of RGB with thermal imaging, represents a promising research direction to enhance model robustness in adverse conditions.

Future work will therefore focus on three key aspects: cross-species generalization to extend the model’s applicability beyond porcine detection, efficient edge deployment to enable real-world implementation, and multi-modal robustness enhancement to ensure reliable performance across diverse environmental conditions. Through these investigations, we aim to transition VM-RTDETR from a powerful proof-of-concept to a versatile and practical solution for intelligent livestock farming.

## 5. Conclusions

This paper proposes a novel pig detection model, VM-RTDETR, to address the critical challenges of global feature capture and multi-scale feature extraction in pig detection. By integrating the innovative VSSD module into the backbone network, our model utilizes a Non-Causal State-Space Duality mechanism to overcome the causal constraints of traditional state-space models, enabling effective modeling of long-range dependencies within images and significantly enhancing the extraction of global structural features of pigs in complex farming environments. Furthermore, the novel Multi-Scale Efficient Hybrid Encoder M-Encoder employs parallel multi-scale convolutional kernels to simultaneously capture local detailed features and global contour information of pigs. This design effectively alleviates the problem of insufficient multi-scale feature extraction caused by varying observation distances and individual size differences among pigs. Compared to traditional models, VM-RTDETR demonstrates significant performance improvements in key metrics: AP increased by 2.35% to 10.93%, $AP_{50}$ improved by 0.63% to 2.82%, and $AP_{75}$ rose by 2.76% to 16.36%. Therefore, the experimental results show that VM-RTDETR provides an efficient and accurate monitoring method for intelligent breeding in animal husbandry.

## Figures and Tables

**Table 1:** Configuration of hardware and software environment for experiments.

Term	Configurations
Operating system	Ubuntu 18.04
GPU	NVIDIA Tesla P100
CPU	Intel(R) Xeon(R) CPU E5-2630 v4 @ 2.20 GHz
GPU environment	CUDA 11.8
Deep learning framework	PyTorch 2.1.1
Compiler	3.10.17
Epochs	100
Memory	128 GB

**Table 2:** Performance comparison of different models.

Model	AP (%)	${\mathrm{AP}}_{50}$ (%)	${\mathrm{AP}}_{75}$ (%)	${\mathrm{AP}}_{m}$ (%)	${\mathrm{AP}}_{l}$ (%)	GFLOPs (G)	Params (M)
Faster R-CNN	35.8	80.9	26.4	24.9	37.6	-	-
YOLOv5n	51.1	91.2	50.2	-	-	7.7	2.6
YOLOv5s	54.7	93.6	55.1	-	-	24.0	9.1
YOLOv6n	51.2	91.3	49.6	-	-	13.0	4.5
YOLOv6s	54.9	93.5	55.3	-	-	44.7	16.4
YOLOv7	59.9	91.2	-	-	-	37.1	105.1
YOLOv8n	52.2	92.1	51.7	-	-	8.7	3.2
YOLOv8s	54.2	92.9	54.4	-	-	28.6	11.2
YOLOv9s	55.7	93.4	56.1	-	-	26.7	7.2
YOLOv12s	52.5	92.0	52.1	-	-	19.4	9.1
YOLOv12n	50.9	91.8	49.4	-	-	6.0	2.5
R50-RTDETR	59.5	94.9	61.6	48.0	61.0	137.7	42.7
**VM-RTDETR**	**60.9**	**95.5**	**63.3**	**50.7**	**62.3**	97.9	28.4

**Table 3:** Performance comparison of the VSSD module.

Model	B	AP (%)	${\mathrm{AP}}_{50}$ (%)	${\mathrm{AP}}_{75}$ (%)	${\mathrm{AP}}_{m}$ (%)	${\mathrm{AP}}_{l}$ (%)	GFLOPs (G)	Params (M)
R18-RTDETR	R18	59.5	94.9	61.7	49.3	60.9	61.1	20.0
R34-RTDETR	R34	59.6	95.2	61.8	49.4	61.1	93.3	31.3
R50-RTDETR	R50	59.5	94.9	61.6	48.0	61.0	137.7	42.7
R101-RTDETR	R101	59.7	95.3	62.1	48.7	61.2	260.6	76.4
VMamba-RTDETR	VMmba	60.2	95.1	62.5	50.0	61.7	87.0	26.3
**V-RTDETR**	VSSD	**60.4**	95.3	**62.9**	50.0	**61.8**	97.9	28.4

**Table 4:** Performance comparison of the M-Encoder module.

Model	B	E	AP (%)	${\mathrm{AP}}_{50}$ (%)	${\mathrm{AP}}_{75}$ (%)	${\mathrm{AP}}_{m}$ (%)	${\mathrm{AP}}_{l}$ (%)	GFLOPs (G)	Params (M)
RTDETR	R50	T	59.5	94.9	61.6	48.0	61.0	137.7	42.7
M-RTDETR	R50	ME	**59.8**	**95.1**	**62.3**	**48.2**	**61.4**	137.7	42.7

**Table 5:** Performance comparison between RTDETR, M-RTDETR, V-RTDETR, and VM-RTDETR models.

Model	B	E	AP (%)	${\mathrm{AP}}_{50}$ (%)	${\mathrm{AP}}_{75}$ (%)	${\mathrm{AP}}_{m}$ (%)	${\mathrm{AP}}_{l}$ (%)	GFLOPs (G)	Params (M)
RTDETR	R50	T	59.5	94.9	61.6	48.0	61.0	137.7	42.7
M-RTDETR	R50	ME	59.8	95.1	62.3	48.2	61.4	137.7	42.7
V-RTDETR	VSSD	T	60.4	95.3	62.9	50.0	61.8	97.9	28.4
**VM-RTDETR**	VSSD	ME	**60.9**	**95.5**	**63.3**	**50.7**	**62.3**	97.9	28.4

## Data Availability

The dataset was developed by our research team and will be made publicly accessible upon reasonable request.
